# Predicting Interactions between Virus and Host Proteins Using Repeat Patterns and Composition of Amino Acids

**DOI:** 10.1155/2018/1391265

**Published:** 2018-05-09

**Authors:** Saud Alguwaizani, Byungkyu Park, Xiang Zhou, De-Shuang Huang, Kyungsook Han

**Affiliations:** ^1^Department of Computer Engineering, Inha University, Incheon 22212, Republic of Korea; ^2^School of Electronics and Information Engineering, Tongji University, Shanghai 201804, China

## Abstract

Previous methods for predicting protein-protein interactions (PPIs) were mainly focused on PPIs within a single species, but PPIs across different species have recently emerged as an important issue in some areas such as viral infection. The primary focus of this study is to predict PPIs between virus and its targeted host, which are involved in viral infection. We developed a general method that predicts interactions between virus and host proteins using the repeat patterns and composition of amino acids. In independent testing of the method with PPIs of new viruses and hosts, it showed a high performance comparable to the best performance of other methods for single virus-host PPIs. In comparison of our method with others using same datasets, our method outperformed the others. The repeat patterns and composition of amino acids are simple, yet powerful features for predicting virus-host PPIs. The method developed in this study will help in finding new virus-host PPIs for which little information is available.

## 1. Introduction

Viral infection involves a large number of protein-protein interactions (PPIs) between virus and its targeted host. These interactions range from the initial binding of viral coat proteins to host membrane receptor to hijack the host transcription machinery by virus proteins. Various viral diseases are caused by infection with pathogenic viruses. For instance, Ebola virus disease is a highly contagious and fatal disease caused by infection with Ebola virus. During the 2014 Ebola epidemic, the world witnessed over 28,000 cases and over 11,000 deaths [1]. So far, there is no specific vaccine or effective treatment for Ebola virus disease [2]. Despite the increased number of known virus-host PPIs, viral infection mechanism is not fully understood. Thus, identifying interactions between virus proteins and host proteins helps understand the mechanism of viral infection and develop treatments and vaccines.

So far, many computational methods have been developed to predict PPIs. However, most of these methods predict PPIs within a single species and cannot be used to predict PPIs between different species because they do not distinguish interactions between proteins of the same species from those of different species. Recently, a few computational methods have been developed to predict virus-host PPIs using machine learning methods. For instance, a homology-based method [3] predicts PPIs between *H. sapiens* and *M. tuberculosis* H37Rv. Support vector machine (SVM) models developed by Cui et al. [4] and Kim et al. [5] predicted PPIs between human and two types of viruses (hepatitis C virus and human papillomavirus). However, these methods are intended for PPIs between virus of a single type and host of a single type. Recent computational methods developed for predicting virus-host PPIs [6–8] are also limited to PPIs between human and the human immunodeficiency virus 1 (HIV-1) and cannot predict PPIs of new viruses or new hosts which have no known PPIs to the methods. A recent SVM model called DeNovo can exceptionally predict PPIs of new viruses with a shared host [9].

In this paper, we present a new method for predicting virus-host PPIs, which is applicable to new viruses or hosts using amino acid repeat patterns and composition. Proteins in a variety of species contain significant amino acid repeats, with more abundance of repeats in eukaryotic proteins than in prokaryotic proteins [10, 11]. It has been found that proteins with a large number of amino acid repeats have a greater number of interacting partners compared to those without [12]. Experimental results of our method show that the repeat patterns and local composition of amino acids are simple, yet powerful features for predicting virus-host PPIs. The rest of this paper discusses the details of the method and its experimental results.

## 2. Materials and Methods

### 2.1. Features and Representation

Proteins are of different lengths and have different amino acid compositions. Many features of proteins have been used to predict PPIs from protein sequences. In this study, we represent a virus-host PPI by three features (F1, F2, and F3):

  F1: sum of squared length of single amino acid repeats (SARs) in the entire protein sequence
  F2: maximum of the sum of squared length of SARs in a window of 6 residues
  F3: composition of amino acids in 5 partitions of the protein sequence

**Figure 1:**
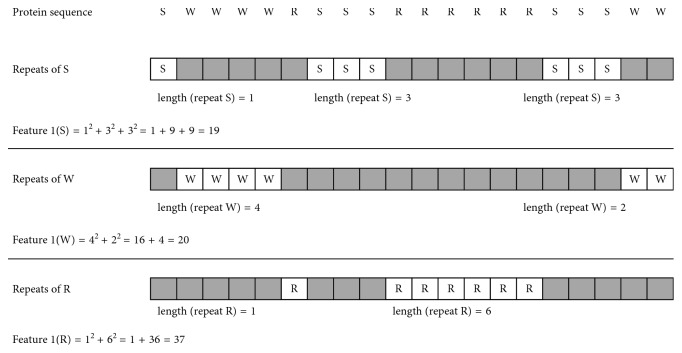
Example of computing feature 1 (F1) of amino acid repeats. F1 is the sum of squared length of single amino acid repeats (SARs) in the whole protein sequence. In this example, F1 (repeats of S) = 1^2^ + 3^2^ + 3^2^ = 19, F1 (repeats of W) = 4^2^ + 2^2^ = 20, and F1 (repeats of R) = 1^2^ + 6^2^ = 37.

F1, which is the sum of squared length of SARs in the protein sequence, is defined by (1). Since SAR of length 1 is also included in F1, the F1 score reflects global composition of amino acids as well as amino acid repeats.  Figure 1 shows an example of how we compute F1.

(1) $$\begin{array}{c} \text{F}1\left(\text{SAR}\right)=\sum_{\text{SAR}\in \text{sequence}}\text{length}{\left(\text{SAR}\right)}^{2}. \end{array}$$

**Figure 2:**
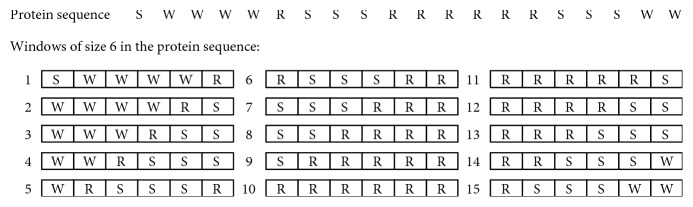
Example of computing feature 2 (F2) of amino acid repeats. F2 is the maximum value of the sum of squared length of single amino acid repeats in a window of size six. The maximum repeat size of amino acid S is 3, which is observed in the windows starting at 4, 5, 6, 7, 13, 14, and 15. So, F2 (repeats of S) = 3^2^ = 9. The maximum repeat size of amino acid W is 4, observed in the windows starting at 1 and 2. F2 (repeats of W) = 4^2^ = 16. The maximum repeat size of amino acid R is 6, observed in the window starting at 10. F2 (repeats of R) = 6^2^ = 36.

Feature F2 is defined by (2). It appears to be similar to F1, but there are two differences: (1) for F2, the sum of squared length of SARs is computed for every window of size 6 instead of a whole protein sequence, and (2) the maximum of the sum of squared length of SARs in a window is selected for F2. For example, a protein sequence SWWWWRSSSRRRRRRSSSWW has 15 possible windows of size 6, as shown in Figure 2. For each amino acid, we compute its F2 score by selecting the maximum of the sum of squared length of the SAR in a window of size 6:

(2) $$\begin{array}{c} \text{F}2\left(\text{SAR}\right)=\underset{\text{window}\in \text{sequence}}{\max}\sum_{\text{SAR}\in \text{window}}\text{length}{\left(\text{SAR}\right)}^{2}. \end{array}$$

**Figure 3:**
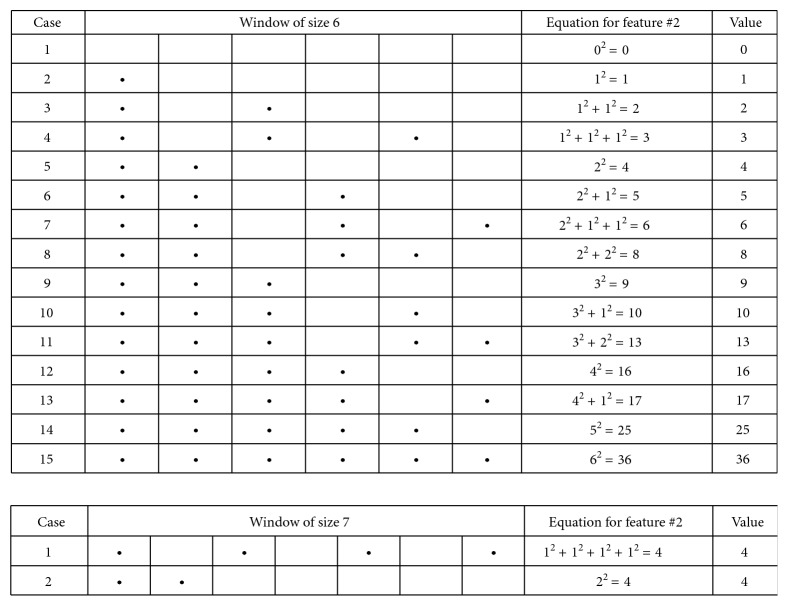
Values of feature 2 for windows of six and seven residues. With a window of size 6, different patterns of single amino acid repeats lead to 15 different values for feature 2. With a window of size 7, different patterns of single amino acid repeats can lead to a same value for feature 2, as shown in this example.

The reason that we use a window of size 6 for F2 is because a window larger than 6 residues generates a same score for different repeat patterns. For example, with a window of size 7, we may obtain a same value of F2 even for different patterns of single amino acid repeats, whereas with a window of size 6, we obtain all different values of F2 for different patterns of single amino acid repeats (Figure 3).

While feature F1 represents the repeat patterns and global composition of amino acids in the whole protein sequence, feature F3 represents the local composition of amino acids. For feature F3, we partition a protein sequence into 5 segments of equal length except the last one and compute the composition of amino acids in each of the 5 segments. Since the three features, F1, F2, and F3, are computed for each amino acid, every pair of virus and host proteins is represented in a feature vector with 280 elements (140 for a virus protein and 140 for a host protein).

### 2.2. Datasets of Virus-Host PPIs

We constructed several datasets of virus-host PPIs to examine the applicability of the prediction method to new viruses and hosts. The first training dataset consists of PPIs of human with positive-sense single-stranded RNA (+ssRNA) viruses except hepatitis C virus (HCV) and severe acute respiratory syndrome (SARS) virus. The SVM model trained with the training dataset was tested on PPIs of five new viruses: HCV, SARS virus, influenza A (H1N1) virus, human papillomavirus (HPV-16), and human immunodeficiency virus HIV-1. Both HCV and SARS are positive-sense single-stranded RNA (+ssRNA) viruses, but the remaining three viruses are of different type. H1N1 virus is a negative-sense single-stranded RNA (−ssRNA) virus, HPV-16 is a double-stranded DNA (dsDNA) virus, and HIV-1 is a retrovirus.

The second training dataset is composed of PPIs between human and +ssRNA viruses, including HCV and SARS virus. The SVM model trained on the second training dataset was tested on PPIs of new hosts: *Mus musculus*, *Bos taurus*, *Rattus norvegicus*, *Sus scrofa*, and *Escherichia coli* K-12.

Data of virus-host PPIs were collected from IntAct [13] and VirusMentha [14]. But PPIs of HCV with human were obtained from the Hepatitis C Virus Protein Interaction Database (HCVpro) [15] because HCVpro has more human-HCV PPIs than IntAct. The sequences of the proteins involved in the virus-host PPIs were obtained from the UniProt database [16].

The training and test datasets constructed in our study can be summarized as follows.

1. Training (TR) and Test (TS) Datasets for Assessing the Applicability of the Prediction Model to New Viruses

  TR1: 638 PPIs between 499 human proteins and 25 +ssRNA virus proteins
  TS1: 515 PPIs between 423 human proteins and 11 HCV proteins
  TS2: 30 PPIs between 27 human proteins and 12 SARS virus proteins
  TS3: 377 PPIs between 307 human proteins and 10 H1N1 virus proteins
  TS4: 319 PPIs between 298 human proteins and 11 HPV-16 proteins
  TS5: 1,578 PPIs between 1,056 human proteins and 46 HIV-1 proteins

2. Training (TR) and Test (TS) Datasets for Assessing the Applicability of the Prediction Model to New Hosts

  TR2: 689 PPIs between 522 human proteins and 35 +ssRNA virus proteins
  TS6: 191 PPIs between 141 *Mus musculus* proteins and 116 virus proteins
  TS7: 125 PPIs between 87 *Bos taurus* proteins and 34 virus proteins
  TS8: 86 PPIs between 79 *Rattus norvegicus* proteins and 24 virus proteins
  TS9: 57 PPIs between 38 *Sus scrofa* proteins and 10 virus proteins
  TS10: 78 PPIs between 64 *Escherichia coli* K-12 proteins and 27 virus proteins

Machine learning-based approaches to PPI prediction require both positive and negative PPI data, but negative data are not available in databases. Constructing a negative dataset of PPIs is not straightforward because there is no experimentally verified noninteracting pair [17]. Eid et al. [9], for example, used negative sampling for their negative dataset. In our study, we constructed a negative dataset with human proteins whose sequence similarity is lower than 40% to any human protein in the positive dataset by running CD-HIT [18]. Our negative dataset includes 2,819 interactions between 90 virus proteins and 2,819 human proteins. The training and test datasets constructed in this study are available in Additional files 1 and 2.

### 2.3. Prediction Models of Virus-Host PPIs

We built several support vector machine (SVM) models using LIBSVM [19] to evaluate our approach. The radial basis function (RBF) was used as a kernel of the SVM models, and the best values of parameters C and *γ* were obtained by running the grid search of LIBSVM on training datasets. Unless specified otherwise, the results shown in this paper were obtained with *C* = 2 and *γ* = 0.5. The SVM models take a pair of virus and host protein sequences as input. As output, the SVM models classify whether or not the virus protein interacts with the host protein. The SVM models and supporting data are available at http://www.bclab.inha.ac.kr/VHPPI.

## 3. Results and Discussion

### 3.1. Performance Measures

The performance of the SVM models was evaluated by several measures: sensitivity (Sn), specificity (Sp), accuracy (Acc), positive predictive value (PPV), negative predictive value (NPV), and Matthews correlation coefficient (MCC), which are defined by the following equations:

(3) $$\begin{array}{c} \text{Sn}=\frac{\text{TP}}{\text{TP}+\text{FN}}, \end{array}$$

(4) $$\begin{array}{c} \text{Sp}=\frac{\text{TN}}{\text{TN}+\text{FP}}, \end{array}$$

(5) $$\begin{array}{c} \text{Acc}=\frac{\text{TP}+\text{TN}}{\text{TP}+\text{FP}+\text{TN}+\text{FN}}, \end{array}$$

(6) $$\begin{array}{c} \text{PPV}=\frac{\text{TP}}{\text{TP}+\text{FP}}, \end{array}$$

(7) $$\begin{array}{c} \text{NPV}=\frac{\text{TN}}{\text{TN}+\text{FN}}, \end{array}$$

(8) $$\begin{array}{c} \text{MCC}=\frac{\left(\text{TP}\times \text{TN}\right)-\left(\text{FP}\times \text{FN}\right)}{\sqrt{\left(\text{TP}+\text{FP}\right)\left(\text{TP}+\text{FN}\right)\left(\text{TN}+\text{FP}\right)\left(\text{TN}+\text{FN}\right)}}. \end{array}$$

In (3)–(8), true positives (TP) are host proteins that are correctly predicted as interacting with a virus protein. True negatives (TN) are noninteracting host proteins that are correctly predicted as noninteracting with a virus protein. False positives (FP) are noninteracting host proteins that are incorrectly predicted as interacting with a virus protein. False negatives (FN) are interacting host proteins that are incorrectly predicted as noninteracting with a virus protein.

### 3.2. Results of Cross Validation

We performed 10-fold cross validation of the SVM model with several datasets which contain different ratios (1 : 1, 1 : 2, and 1 : 3) of positive to negative PPIs between +ssRNA viruses and human. As shown in Table 1, the best performance of the SVM model was observed in the balanced dataset with 1 : 1 ratio of positive to negative data. As expected, running the SVM model on unbalanced datasets resulted in lower performances than running it on the balanced dataset with 1 : 1 ratio of positive to negative data. Datasets are available in Additional file 3.

We also examined the contribution of the features to the prediction performance of the SVM model.  Table 2 shows the results of using different combinations of features in 10-fold cross validation of the SVM model with the 1 : 1 dataset of Table 1. Among the single features, F3, which is the local composition of amino acids, was the best in all performance measures. With F3 alone, the SVM model achieved an accuracy above 92% and an MCC above 0.86, indicating that F3 is a very powerful feature in predicting virus-host PPIs. The best performance of the SVM model was observed when F1 and F3 were used. We also examined this work with different combinations of features. We used double amino acid repeats (DARs) for F1 and F2 instead of single amino acid repeats (SARs), but here for F2, we used a window size of 10 residues not 6 residues because we are working with DAR, so a window size of 10 residues is the biggest available window size that obtain a different value for every double amino acid repeat in it, but a window size of 6 residues does the same thing for the single amino acid repeat.

For features F1 and F2, we tried both single amino acid repeats (SARs) and double amino acid repeats (DARs) along with different partitions of a protein sequence. As shown in Table 3, SAR resulted in a better performance than DAR.

For feature F3, we tried several different partitions of a protein sequence in several datasets.  Table 4 shows the performance of our SVM model in three different datasets of virus-host PPIs. All the results shown in Table 4 were obtained by using SAR for features F1 and F2, but with different partitions for feature F3. On average, partitioning a protein sequence into 5 segments showed the best performance in all performance measures except sensitivity. In addition to the performance gain, partitioning a protein sequence into 5 segments is more advantageous than 7 or 9 segments with respect to the size of a feature vector that represents the sequence. When we partition a protein sequence into 5 segments, every pair of virus and host proteins is encoded in a feature vector with 280 elements (20 elements for F1, 20 elements for F2, and 20 × 5 = 100 elements for F3 for each of the virus and host proteins). If we partition a protein sequence into 7 or 9 partitions, a feature vector will require 360 elements (20 elements for F1, 20 elements for F2, and 20 × 7 = 140 elements for F3 for each of the virus and host proteins) or 440 elements (20 elements for F1, 20 elements for F2, and 20 × 9 = 180 elements for F3 for each of the virus and host proteins). However, the larger feature vectors did not result in performance improvement in predicting virus-host PPIs.

### 3.3. Results of Independent Testing on PPIs of New Viruses

As discussed earlier, we trained the SVM model with the training dataset TR1 consisting of PPIs of human with +ssRNA viruses except hepatitis C virus (HCV) and SARS virus and tested it on PPIs of new viruses which were not used in training the model. The test datasets include PPIs of five viruses (HCV, SARS virus, H1N1 virus, HPV-16, and HIV-1) with human. H1N1 virus is a negative-sense single-stranded RNA (-ssRNA) virus, and HPV-16 is a double-stranded RNA (dsDNA) virus. HIV-1 is a retrovirus, which is a +ssRNA virus with a DNA intermediate.

In addition to species difference, we also analyzed the sequence similarity between the training datasets and test datasets using EMBOSS Needle tool [20] to assess the independence of the test data from the training data. As shown in Table 5, target virus proteins in the test datasets showed a very low average sequence similarity in the range (3.12% to 5.20%) to the virus proteins in the training dataset (see Additional file 4 for the similarity of every sequence pair between the training and test datasets).

**Figure 4:**
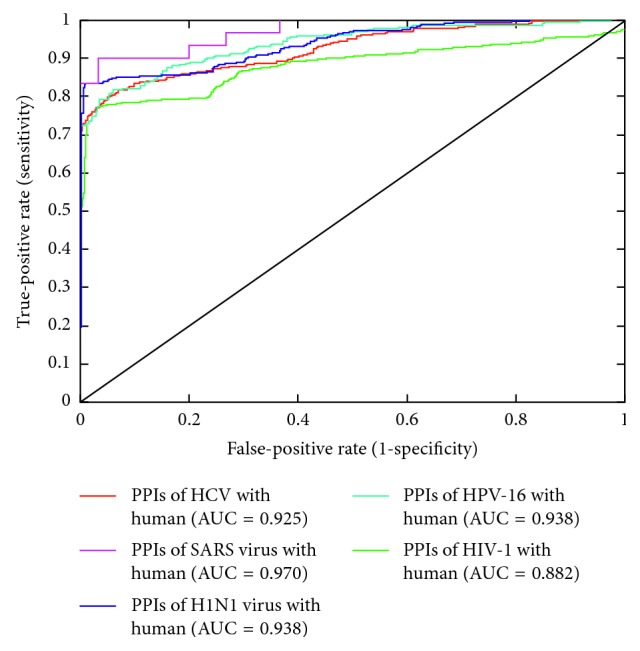
ROC curves of independent testing of the SVM model on PPIs of new viruses with human. The SVM model showed the largest area under the ROC curve (AUC) of 0.970 for the PPIs of the SARS virus with human.

Table 6 shows the results of testing the prediction model on 5 independent datasets of PPIs of new viruses. Despite such a low sequence similarity and species difference, the SVM model showed a high performance in independent testing. In particular, the SVM model showed a higher sensitivity (94.37% and 96.67%) for HCV and SARS virus, which are +ssRNA viruses. It is interesting to note that HPV-16, which is a dsDNA virus, showed the highest specificity of 94.04% and accuracy of 87.93%.  Figure 4 shows the ROC curves of independent testing of the SVM model on PPIs of five new viruses.

### 3.4. Results of Independent Testing on PPIs of New Hosts

**Figure 5:**
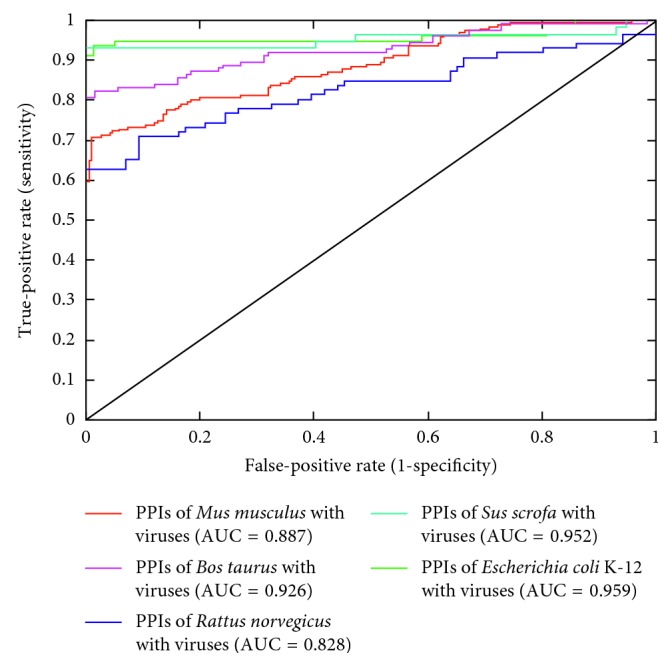
ROC curves of independent testing of the SVM model on PPIs of new hosts with viruses. The SVM model showed the largest area under the ROC curve (AUC) of 0.959 for the PPIs of *E. coli* K-12 with viruses.

In order to examine the applicability of the SVM model to new hosts, we tested it on PPIs of viruses with new hosts, which were not used in training the model. As described earlier, the model trained with PPIs of human with +ssRNA viruses was tested on PPIs of five new hosts (*Mus musculus*, *Bos taurus*, *Rattus norvegicus*, *Sus scrofa*, and *Escherichia coli* K-12) with the viruses. As shown earlier in Table 5, the average sequence similarity of the human proteins in the training dataset to the new hosts is low, ranging between 8.04% and 9.76%. Despite the low sequence similarity and species difference, testing the model on PPIs of new hosts showed a relatively good performance (Table 7).  Figure 5 shows the ROC curves of independent testing of the SVM model on PPIs of five new hosts.

It is interesting to note that proteins of new hosts have a higher average sequence similarity to those in training datasets than proteins of new viruses, but the SVM model showed a lower performance for new hosts. This can be explained by the number of partner proteins of the target proteins shared by training and test datasets. As shown in Table 8, the number of common proteins between the test datasets for new viruses (TS1-TS5) and their training dataset TR1 is larger than the number of common proteins between the test datasets for new hosts (TS6-TS10) and their training dataset TR2. Thus, the SVM model showed a better performance for new viruses than for new hosts. These results corroborate the known problem with pair-input methods, which was first reported by Park and Marcotte [21]. According to their study [21], prediction methods that operate on pairs of objects such as PPIs perform much better for test pairs that share components with a training set than for those that do not. Thus, our prediction model showed a better performance in testing for new viruses which share more partner proteins (i.e., host proteins) with training datasets than in testing for new hosts which share fewer partner proteins (i.e., virus proteins) with training datasets.

### 3.5. Comparison to Other Methods

For a comparative purpose, we ran our SVM model on the datasets of two other methods for virus-host PPIs: Barman's method [22] and DeNovo [9]. In Barman's study [22], three machine learning methods (SVM, Naive Bayes, and Random Forest) were used to predict virus-host PPIs using several features such as domain -domain association in interacting protein pairs and composition of methionine, serine, and valine in virus proteins. In a 5-fold cross validation with virus-host PPIs from VirusMINT [23], their Random Forest (RF) and SVM showed a better performance than Naive Bayes. Thus, we tested our SVM model on the same dataset used in Barman's study, which contains 1,035 positive and 1,035 negative interactions between 160 virus proteins of 65 types and 667 human proteins. As shown in Table 9, our SVM model outperformed Barman's SVM model in all performance measures and our SVM model outperformed Barman's RF model in all performance measures except specificity and PPV. The dataset used for comparison of our SVM model with Barman's SVM and Random Forest models is available in Additional file 5.

For comparison with DeNovo's SVM model, we tested our SVM model on DeNovo's SLiM testing set, which contains 425 positive and 425 negative PPIs (Supplementary file S12 used in DeNovo's study ST6). As shown in Table 10, our SVM model was better than DeNovo in all performance measures (sensitivity of 86%, specificity of 87%, and accuracy of 86%). The dataset used for comparison of our SVM model with DeNovo is available in Additional file 6.

## 4. Conclusions

Amino acid repeats are prevalent in a variety of proteins but are rarely used in predicting PPIs. We developed a new method that predicts potential interactions between virus and host proteins using global and local compositions of amino acids as well as amino acid repeat patterns.

We tested the prediction model on independent datasets of virus-host PPIs, which were not used in training the model and have a very low sequence similarity to any protein in training datasets of the model. Despite a low sequence similarity between proteins in training datasets and target proteins in test datasets, the prediction model showed a high performance comparable to the best performance of other methods for single virus-host PPIs. In comparison of our method with others using same datasets, our method outperformed the others. Experimental results demonstrate that the repeat patterns and composition of amino acids are simple, yet powerful features for predicting virus-host PPIs. The method can be used to find potential PPIs of new viruses or hosts, for which little information is known.

## Figures and Tables

**Table 1:** Results of 10-fold cross validation of SVM model on 1,072 PPIs between 36 RNA viruses and 812 human proteins with different ratios of positive to negative instances.

P : N	Dataset	Sn (%)	Sp (%)	Acc (%)	PPV (%)	NPV (%)	MCC	AUC
1 : 1	1	88.24	97.34	92.79	97.07	89.22	0.859	0.963
	2	81.03	94.36	87.7	93.49	83.26	0.761	0.931
	3	77.74	94.04	85.89	92.88	80.86	0.728	0.926
	mean ± SD	82.34 ± 4.39	95.25 ± 1.49	88.79 ± 2.92	94.48 ± 1.85	84.45 ± 3.51	0.78 ± 0.06	0.94 ± 0.02

1 : 2	1	64.89	97.34	86.52	92.41	84.72	0.693	0.893
	2	58.31	97.57	84.48	92.31	82.4	0.646	0.886
	3	63.64	96.08	85.27	89.04	84.09	0.661	0.891
	mean ± SD	62.28 ± 2.85	97 ± 0.65	85.42 ± 0.84	91.25 ± 1.57	83.74 ± 0.98	0.67 ± 0.02	0.89 ± 0.00

1 : 3	1	46.24	98.28	85.27	89.94	84.58	0.58	0.850
	2	46.87	98.59	85.66	91.72	84.77	0.59	0.863
	3	49.37	97.28	85.31	85.83	85.22	0.576	0.858
	mean ± SD	47.49 ± 1.35	98.05 ± 0.56	85.41 ± 0.18	89.16 ± 2.47	84.86 ± 0.27	0.58 ± 0.01	0.86 ± 0.01

Sn: sensitivity, Sp: specificity, Acc: accuracy, PPV: positive predictive value, NPV: negative predictive value, MCC: Matthews correlation coefficient, and AUC: the area under the ROC curve.

**Table 2:** Comparison of different combinations of features in 10-fold cross validation of SVM model.

Features	Sn (%)	Sp (%)	Acc (%)	PPV (%)	NPV (%)	MCC	AUC
F1	81.66	97.02	89.34	96.48	84.10	0.796	0.916
F2	69.75	85.11	77.43	82.41	73.78	0.555	0.849
F3	87.78	97.81	92.79	97.56	88.89	0.860	0.965
F1 + F2	80.88	95.61	88.24	94.85	83.33	0.773	0.925
F1 + F3	88.56	97.34	92.94	97.08	89.48	0.862	0.966
F2 + F3	87.46	96.87	92.16	96.54	88.54	0.847	0.961
F1 + F2 + F3	88.24	97.34	92.79	97.07	89.22	0.859	0.963

F1: sum of squared length of single amino acid repeats in the entire protein sequence, F2: maximum of the sum of squared length of single amino acid repeats in a window of 6 residues, F3: composition of amino acids in 5 partitions of the protein sequence, Sn: sensitivity, Sp: specificity, Acc: accuracy, PPV: positive predictive value, NPV: negative predictive value, MCC: Matthews correlation coefficient, and AUC: the area under the ROC curve.

**Table 3:** Results of 10-fold cross validation of SVM model on different combinations of the three features we used in our method.

F1 and F2	F3	Sn (%)	Sp (%)	Acc (%)	PPV (%)	NPV (%)	MCC	AUC
SAR	5 partitions	88.24	97.34	92.79	97.07	89.22	0.859	0.963
SAR	7 partitions	88.24	97.96	93.10	97.74	89.29	0.866	0.965
SAR	9 partitions	89.19	96.08	92.63	95.79	89.88	0.855	0.962
DAR	5 partitions	84.80	94.51	89.66	93.92	86.14	0.797	0.937
DAR	7 partitions	85.42	94.51	89.97	93.97	86.64	0.803	0.938
DAR	9 partitions	85.27	94.20	89.73	93.63	86.47	0.798	0.940

SAR: single amino acid repeats for F1 and F2, DAR: double amino acid repeats for F1 and F2, Sn: sensitivity, Sp: specificity, Acc: accuracy, PPV: positive predictive value, NPV: negative predictive value, MCC: Matthews correlation coefficient, and AUC: the area under the ROC curve.

**Table 4:** Results of testing our SVM model with different partitions of a protein sequence on three datasets.

*Our dataset*							
F3	Sn (%)	Sp (%)	Acc (%)	PPV (%)	NPV (%)	MCC	AUC
5 partitions	88.24	97.34	92.79	97.07	89.22	0.859	0.963
7 partitions	88.24	97.96	93.10	97.74	89.29	0.866	0.965
9 partitions	89.19	96.08	92.63	95.79	89.88	0.855	0.962

*DeNovo dataset*							
F3	Sn (%)	Sp (%)	Acc (%)	PPV (%)	NPV (%)	MCC	AUC
5 partitions	86.35	86.59	86.47	86.56	86.39	0.729	0.926
7 partitions	83.60	81.18	82.41	82.30	82.54	0.648	0.907
9 partitions	84.27	79.53	81.95	81.17	82.84	0.639	0.902

*Barman dataset*							
F3	Sn (%)	Sp (%)	Acc (%)	PPV (%)	NPV (%)	MCC	AUC
5 partitions	73.72	83.48	78.60	81.69	76.06	0.575	0.847
7 partitions	78.55	78.55	78.55	78.55	78.55	0.571	0.858
9 partitions	78.16	79.81	78.99	79.47	78.52	0.580	0.860

*Average of the above three results*							
F3	Sn (%)	Sp (%)	Acc (%)	PPV (%)	NPV (%)	MCC	AUC
5 partitions	82.77	89.14	85.95	88.44	83.89	0.721	0.912
7 partitions	83.46	85.90	84.69	86.20	83.46	0.695	0.910
9 partitions	83.87	85.14	84.52	85.48	83.75	0.691	0.908

All the results were obtained by commonly using SAR for features F1 and F2.

**Table 5:** Training (TR) and test (TS) datasets for assessing the applicability of the SVM model to new viruses and to new hosts. The average sequence similarity between proteins in TR and those in TS was analyzed using EMBOSS Needle tool [20].

Proteins in training datasets	Target proteins in test datasets	Average sequence similarity (%)
25 virus proteins in TR1	11 HCV proteins in TS1	5.03
	12 SARS virus proteins in TS2	5.20
	10 H1N1 virus proteins in TS3	5.03
	11 HPV-16 proteins in TS4	3.12
	46 HIV-1 proteins in TS5	3.56

522 human proteins in TR2	141 *Mus musculus* proteins in TS6	9.20
	87 *Bos taurus* proteins in TS7	9.07
	79 *Rattus norvegicus* proteins in TS8	9.76
	38 *Sus scrofa* proteins in TS9	8.70
	64 *Escherichia coli* K-12 proteins in TS10	8.04

**Table 6:** Results of independent testing our SVM on PPIs of new viruses with human.

Virus	Sn (%)	Sp (%)	Acc (%)	PPV (%)	NPV (%)	MCC	AUC
HCV	94.37	52.04	73.20	66.30	90.24	0.512	0.925
SARS virus	96.67	73.33	85.00	78.38	95.65	0.720	0.970
H1N1 virus	90.72	67.90	79.31	73.87	87.97	0.602	0.938
HPV-16	81.82	94.04	87.93	93.21	83.80	0.764	0.938
HIV-1	87.83	64.64	76.24	71.30	84.16	0.539	0.882
Average	90.28	70.39	80.34	76.61	88.36	0.627	0.930

Sn: sensitivity, Sp: specificity, Acc: accuracy, PPV: positive predictive value, NPV: negative predictive value, MCC: Matthews correlation coefficient, and AUC: the area under the ROC curve.

**Table 7:** Results of independent testing our SVM on PPIs of new hosts with viruses.

Host	Sn (%)	Sp (%)	Acc (%)	PPV (%)	NPV (%)	MCC	AUC
*Mus musculus*	85.86	61.78	73.82	69.20	81.38	0.491	0.887
*Bos taurus*	98.40	27.20	62.80	57.48	94.44	0.365	0.926
*Rattus norvegicus*	91.86	27.90	59.88	56.03	77.42	0.257	0.828
*Sus scrofa*	100.00	5.26	52.63	51.35	100.00	0.164	0.952
*Escherichia coli* K-12	94.87	91.03	92.95	91.36	94.67	0.860	0.959
Average	92.02	54.23	73.13	67.80	86.86	0.501	0.911

Sn: sensitivity, Sp: specificity, Acc: accuracy, PPV: positive predictive value, NPV: negative predictive value, MCC: Matthews correlation coefficient, and AUC: the area under the ROC curve.

**Table 8:** The number of host proteins shared by training (TR) and test (TS) datasets used for assessing the applicability of the SVM model to new viruses and to new hosts.

Dataset	TR1	TS1	TR1	TS2	TR1	TS3	TR1	TS4	TR1	TS5
#PPIs	638	515	638	30	638	377	638	319	638	1578
#Virus proteins	25	11	25	12	25	10	25	11	25	46
#Host proteins	499	424	499	27	499	307	499	298	499	1056
#Host proteins common to TR and TS	63 (14.9%)		5 (18.5%)		68 (22.1%)		22 (7.4%)		122 (11.6%)	

Dataset	TR2	TS6	TR2	TS7	TR2	TS8	TR2	TS9	TR2	TS10
#PPIs	689	191	689	125	689	86	689	57	689	78
#Virus proteins	35	116	35	34	35	24	35	10	35	27
#Host proteins	522	141	522	87	522	79	522	38	522	64
#Virus proteins common to TR and TS	9 (7.8%)		1 (2.9%)		4 (16.7%)		0 (0.0%)		0 (0.0%)	

The numbers in parentheses represent the proportion of common proteins to proteins in test datasets.

**Table 9:** Results of 5-fold cross validation of our SVM and Barman's SVM [22] with Barman's dataset of 1,035 positive and 1,035 negative PPIs.

Method	Sn (%)	Sp (%)	Acc (%)	PPV (%)	NPV (%)	MCC	AUC	F1 (%)
Our SVM	73.72	83.48	78.60	81.69	76.06	0.575	0.847	77.50
Barman's SVM	67.00	74.00	71.00	72.00	—	0.440	0.730	69.41
Barman's Random Forest	55.66	89.08	72.41	82.26	—	0.480	0.760	66.39

Sn: sensitivity, Sp: specificity, Acc: accuracy, PPV: positive predictive value, NPV: negative predictive value, MCC: Matthews correlation coefficient, AUC: the area under the ROC curve, F1 = 2 × (SN × PPV)/(SN + PPV), and “—”: not available.

**Table 10:** Results of testing our SVM and DeNovo's SVM [9] on DeNovo's dataset of 425 positive and 425 negative PPIs.

Method	Sn (%)	Sp (%)	ACC (%)	PPV (%)	NPV (%)	MCC	AUC
Our SVM	86.35	86.59	86.47	86.56	86.39	0.729	0.926
DeNovo's SVM	80.71	83.06	81.90	—	—	—	—

Sn: sensitivity, Sp: specificity, Acc: accuracy, PPV: positive predictive value, NPV: negative predictive value, MCC: Matthews correlation coefficient, AUC: the area under the ROC curve, and “—”: not available.

## Supplementary Materials

Supplementary Materials The supporting data and program are available at http://www.bclab.inha.ac.kr/VHPPI. Additional file 1: training and test datasets for examining the applicability of the SVM model to new viruses. Additional file 2: training and test datasets for examining the applicability of the SVM model to new hosts. Additional file 3: training datasets with different ratios of positive to negative instances (1 : 1, 1 : 2, and 1 : 3). Additional file 4: the sequence similarity of each protein pair (*x*, *y*) for *x* in the training dataset and *y* in the test dataset. Additional file 5: training and test datasets of virus-host PPIs from Barman's study [22]. Additional file 6: training and test datasets of SLiMs from DeNovo [9].
